# Hybrid Mode Sensor Fusion for Accurate Robot Positioning

**DOI:** 10.3390/s25103008

**Published:** 2025-05-10

**Authors:** Viktor Masalskyi, Andrius Dzedzickis, Igor Korobiichuk, Vytautas Bučinskas

**Affiliations:** 1 Department of Mechatronics, Robotics and Digital Manufacturing, Vilnius Gediminas Technical University, LT-10105 Vilnius, Lithuaniaandrius.dzedzickis@vilniustech.lt (A.D.); 2 Institute of Automatic Control and Robotics, Warsaw University of Technology, 02-525 Warsaw, Poland

**Keywords:** sensor signal fusion, robotics, robot positioning, hybrid mode, fusion software

## Abstract

**Highlights:**

**What are the main findings?**

- Sensor data fusion works—simpler sensors have higher accuracy.
- There is an increase in performance with sensor fusion in a hybrid mode compared to a single-level fusion process.

**What is the implication of the main finding?**

- Robotic positions can be rectified using sensor data fusion.
- Typically, a sensor fusion level is focused at the third level of fusion according to the provided classification.

**Abstract:**

Robotic systems are becoming increasingly crucial in applications requiring high precision. While a robot can operate using basic sensor feedback under controlled conditions, achieving micro-level accuracy requires more comprehensive data integration, especially in dynamic environments. The fusion of data from a variety of sensors is necessary for improving the positioning accuracy of a robot because the accuracy of one type of sensor is insufficient. The field of micro-positioning presents new challenges and tasks that have been gradually explored in the recent literature published from 2015 to 2025. Micro-positioning is a complex operation that involves factors such as mechanical drift, environmental effects, and sensor signal errors. Hybrid fusion is a sensor fusion technique that combines elements of fusion at different levels. For the effective deployment of robots in such contexts, it is essential to integrate multiple sensors and ensure reliable data fusion between them. This involves the use of different sensors, advanced fusion algorithms, and accurate calibration methods through sensor fusion and sophisticated data processing techniques. This literature review presents an analysis of the sensor data fusion methods for precise robot micro-positioning. The focus is on the investigated sensors, the applied synthesis methods, and the developed algorithms and their practical application to identify the existing gaps for future system improvements. Finally, discussions and conclusions based on the collected ideas are presented.

## 1. Introduction

One of the key challenges in the microrobot area is optimizing the methods for fusion of data from different sensors. A main component to efficient operation in a variety of fields, including automation, artificial intelligence, robotics, and microelectromechanical systems (MEMSs), is precise positioning in multiaxial systems [1]. These systems are required to execute tasks with great precision and consistency since they operate along multiple axes. Positioning errors can result in emergencies, poor efficiency, and even flawed products. Securing precise and dependable positional data for each axis is among the most significant challenges when dealing with multi-axis systems. Numerous elements such as mechanical backlash, vibration, thermal deformation, and external influences make this difficult. Individual sensors such as optical sensors, encoders, or inertial measurement units (IMUs) have limitations and do not always provide the required accuracy on their own [2]. The authors emphasize the importance of improving sensor technologies and sensor fusion algorithms to achieve higher levels of autonomy and safety.

Different sensors, such as temperature sensors, are prone to accumulating errors over time due to minor inaccuracies in measurements, resulting in what is known as “drift”, which can greatly distort positional data in short durations [3]. The main goal of one study was to build a thermal deformation prediction model using an artificial neural network and implement real-time error compensation. Optical sensors, including laser rangefinders and cameras, deliver high precision and resolution, but are influenced by environmental conditions. Integrating data from various sensor types, or multi-sensor integration, helps to overcome these constraints. When using data from IMUs and optical sensors, it is possible to leverage the strengths of each. The IMUs provide high-frequency data with low latency and the optical sensors provide high-precision information to correct IMU errors [4].

Robots equipped with combined sensor systems can position the end-of-arm tool or gripper with minimal error. One study merged data from different sensors, which reduced the robot positioning error to several micrometers, which significantly increased the accuracy of its movements [5]. The developed system allowed for the early detection of defects, which can lead to increased production efficiency and reduced costs, but it was not fully automated. During the automated welding of large structures, it is important to consider deformations of the material, but the system requires significant calibration overhead that limits deployment flexibility. Using a combination of sensors to correct inspection trajectories in real time could ensure the production of high-quality welded seams. Research has indicated an enhancement in welding accuracy thanks to multi-sensor integration [6].

Machining parts with a complex shape, such as implants, using computer numerical control (CNC) machines requires the coordination of movement along several axes. The use of combined data from angular encoders, linear sensors, and vibration sensors makes it possible to compensate for dynamic loads and vibrations, increasing the processing accuracy.

The leading machine tool manufacturers use systems that combine data from different sensors to optimize processes [7]. Fluctuations in temperature can lead to the expansion or contraction of CNC machine components, affecting precision. Integrating data from temperature and position sensors facilitates real-time adjustment of positional parameters. A recent study showed that employing a temperature and position sensor data fusion approach greatly reduces positioning errors [8].

In smartphones, cameras use MEMS actuators to counteract hand tremors. Combining data from gyroscopes and optical sensors allows for the rapid adaptation to motion and maintenance of image clarity. According to research, such systems can reduce image blurring and improve picture quality [9]. In drones and portable devices, MEMS sensors are often used for the generation of orientation and stabilization signals, which is further used to control actuators. However, due to miniaturization, drones and portable devices are lighter; therefore, noise and environmental vibrations create more disturbances. Using algorithms such as the extended Kalman filter and integrating data from accelerometers, gyroscopes, and magnetometers can enhance navigation accuracy. Studies have shown that multi-sensory integration in MEMS devices results in a reduction in orientation errors [10].

Producing parts that possess intricate geometry and detailed components using 3D printers requires precise control of positioning along all axes. A high accuracy in directing the extrusion head is achieved through the integration of data from stepper motors, linear encoders, and optical sensors. To maintain the consistency of the first layer, a critical factor in overall quality of the product, some companies have adopted active leveling systems for print platforms that use data from various sensors [11].

The integration of data from optical sensors, accelerometers, and infrared (IR) sensors allows for the monitoring of process parameters in real time, preventing defects and ensuring printing quality, i.e., it reduces the number of printing defects per plane [12].

Sensor fusion technology is used in invasive surgery as well as instrument movement should have extreme precision in this setting. Typically, by combining sensor signal data from tactile sensors, optical trackers, and inertial sensors, haptic instrument control generates the required feedback to the operating surgeon. Well-known systems such as the da Vinci surgical system use multi-sensory data to filter hand tremors and scale movements, increasing the safety and efficiency of operations [13].

Opting for the appropriate fusion method can greatly enhance the precision and efficiency of systems in different industries. An illustrative example is the incremental training of long short-term memory (LSTM) autoencoder models to detect anomalies in CNC machine tools. Ref. [14] showed how such technology allows models to adjust to changing operating conditions, which is crucial for the rapid detection and prevention of real-time failures. Ref. [15] describes methods that can prolong the life of cutting tools and reduce maintenance costs by implementing artificial neural networks and integrating data from various sensors.

Equipment fault diagnosis also greatly benefits from the use of multi-sensor data fusion techniques. The same methods allow faults to be detected and corrected in a timely manner, thus improving the reliability and safety of production processes [16]. The use of modern deep learning and data fusion methods is not limited to industry. This article discusses various approaches that can significantly improve the detection and diagnosis of problems in building structures, contributing to more efficient maintenance and increased service life of objects.

The integration of sensor data fusion with artificial intelligence (AI) and machine learning (ML) is gaining importance due to the rising complexity of data across various fields. Sensor data fusion merges inputs from different sensors to improve precision and reliability, while AI and ML provide strong tools for analyzing this combined data.

Combining various sensors, like IMUs, radar, and software-defined radios, has greatly improved Human Activity Recognition (HAR) [17], overcoming the constraints of single-sensor systems. Inspired by the human brain, neuromorphic computing has excelled in accurately identifying specific actions with minimal data. Moreover, hybrid deep learning architectures that merge CNNs, LSTM, and self-attention mechanisms have attained remarkable precision in interpreting data from wearable sensors [18].

The extensive connectivity of IoT networks, particularly in industrial settings, presents intricate cybersecurity issues [19]. AI and ML, with an emphasis on anomaly detection paired with sensor fusion, bolster system resilience by effectively spotting faults and threats. Techniques like particle filters and algorithms such as XGBoost [20] have proven successful in protecting critical infrastructure from cyber threats and operational issues.

The combination of AI, ML, and sensor data is revolutionizing healthcare by facilitating more personalized and proactive approaches. Methods like Multi-Armed Bandit that employ multi-sensor setups have been used for precise sleep monitoring in the elderly [21]. Digital Twins and integrated sensor-conversational AI systems deliver advanced, customized insights and predictive abilities, significantly improving healthcare outcomes [22].

Sensor data fusion is also used for Earth observations. Earth observations increasingly depend on fusing multi-modal data like Synthetic Aperture Radar (SAR) and multispectral imagery for thorough environmental monitoring. Machine learning algorithms can adeptly handle and analyze the complexity and scale of these datasets, although challenges persist due to the limited availability of ML-ready SAR datasets. Initiatives to tackle these issues, such as the development of the M3LEO dataset, mark the notable progress in AI-powered Earth observation technologies [23].

In addition, the researchers in [24] focused on applying data fusion techniques to improve precision and efficiency in CNC machining, especially in the development of nanocomposites. The consolidation of data from multiple sensors significantly improved the control of the process and the quality of the final products. Combining sensor data and advanced analytical techniques aided in boosting the efficiency and reliability of the mechanical production processes. Regardless of the specific application (in various CNC machine tools, structural health monitoring, or predictive fault diagnosis), deploying data fusion and machine learning increases productivity and ensures technological security. One study reviewed several SFAs, including various Kalman filter modifications, compared to a camera motion-capture system. SFA gains were enhanced through particle swarm optimization and effective techniques to minimize estimation errors were identified, particularly during extended trials. This analysis of sensor fusion algorithms (SFAs) for 3D orientation tracking employed magnetic and inertial measurement units (MIMUs). The objective of the study was to contribute to establishing a comprehensive online repository for SFAs. To improve positioning precision, a sensor fusion framework was suggested for indoor localization utilizing smartphone inertial measurement unit (IMU) sensors and Wi-Fi received signal strength indication (RSSI) measurements [25]. This framework integrates Wi-Fi location fingerprinting, trilateration, and pedestrian dead reckoning (PDR) to refine positioning accuracy, and achieved a maximum localization error of 1.17 m. In [26], an adaptive heterogeneous fusion algorithm was introduced for real-time processing to enhance the fusion of gyroscope, accelerometer, and magnetometer data for orientation and heading estimation. The proposed algorithm achieved a faster dynamic response compared to the extended Kalman filter (EKF), while requiring less computational time. In addition, it supports a novel real-time calibration method using machine learning to compensate for sensor thermal drift.

A proposed event-based multi-sensor fusion algorithm effectively handled dead zone measurements by only transmitting significant data to the fusion host in [27]. This approach uses a modified Kalman filter (KF) and its information form to manage the dead zone-like measurements. The simulation results showed that the algorithm offers a good trade-off between performance and communication bandwidth.

Ref. [28] outlines the obstacles faced in indoor localization due to the blockage of Global Navigation Satellite System (GNSS) signals and stresses the importance of using independent localization techniques such as odometry and simultaneous localization and mapping (SLAM). It examined different sensors like Light Detection and Ranging (LiDAR), IMUs, radar, and cameras along with their uses, covering the algorithms and fusion frameworks for indoor odometry, while also discussing future opportunities.

Failures and inaccuracies in sensor measurements, particularly in autonomous systems that are highly dependent on sensors, can have significant repercussions [2,25]. There is a need for more durable sensors, as well as failure detection and compensation, to solve these issues [2,26]. Combining data from diverse sensors, particularly those with varying levels of accuracy and frequency, remains a difficult undertaking. More effective fusion algorithms that consider data uncertainty and redundancy are necessary. Proper sensor calibration and synchronization are critical for the precise functioning of multi-sensory systems [26,27], which is particularly vital in real-time applications. Multi-sensory systems should be strong enough to withstand noise and data errors [26]. Techniques need to be devised to ensure the reliability of the models in real-world scenarios [29,30].

This review effectively demonstrates the advantages of integrating multiple sensors across various fields, such as robotics, CNC machining [8], surgical systems [31], and indoor localization [25], by emphasizing how combining sensors like IMUs, optical devices, and encoders greatly enhances precision, reduces errors, and increases reliability. It highlights advanced techniques like Kalman filtering, neural networks, and incremental LSTM autoencoders [14], showcasing their flexibility, real-time performance, and computational efficiency. While the benefits like improved accuracy and fault detection are well known, the analysis would be strengthened through more explicit comparative benchmarks, a clearer identification of the methods’ limitations, and additional validation details to boost the overall analytical strictness and practical applicability.

The aim of this review is to analyze the existing methods of positioning error compensation that are suitable for micrometer-scale manipulation processes and in microrobotics using different sensor fusion methods.

The layout of the paper is organized as follows. Section 3 explores the technology behind sensor fusion, detailing the integration of information from multiple sensors across various levels (data, feature, and decision levels) and examines popular software frameworks for fusion. Section 4 details the specific applications of sensor fusion in mobile robotics, manufacturing processes, and specialized fusion algorithms, alongside localization and position detection. Section 5 provides a thorough analysis of the core results, emphasizing the key trends in adopting hybrid data fusion. Section 6 concludes the paper by highlighting the main outcomes and proposing possible directions for future research.

## 2. Materials and Methods

**Figure 1:**
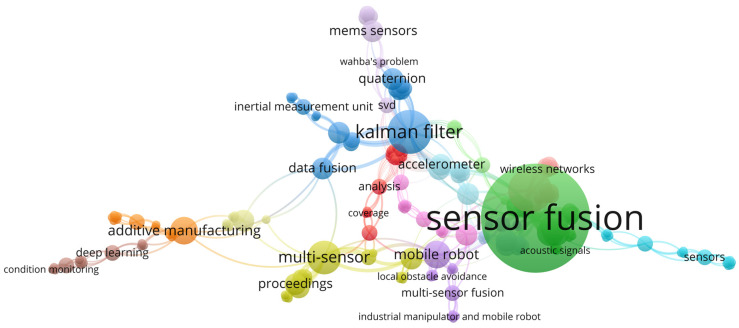
Bibliographic network of research keyword distribution.

This paper was prepared using different science databases such as the Google Scholar, MDPI, IEEE Xplore, Web of Science, and SCOPUS bibliographic databases. Semantic Scholar and an AI-powered research tool were used for the bibliographic research and analysis. The following keywords were used: “sensor fusion”, “CNC machines”, “3D printers”, “smartphones”, “production PCB”, “minimal invasive surgery”, “microposition”, “sensor fusion algorithms”. A bibliographic map of the selected studies are graphically shown in Figure 1.

The studies were selected based on the following:

- Titles;
- Research results;
- Methodology;
- Applications.

The keywords were chosen to cover a wide range of sensor fusion research in which sensor technologies were applied in areas from industrial production to medical research.

During the investigation, 180 articles were obtained, of which, 104 publications were considered valuable and high-quality papers and were selected for analysis. The inclusion criteria included a clear methodology and significant results with validation. This paper includes articles published from 2015 to 2025, which allowed us to cover new achievements and approaches in this field.

## 3. Technology of Sensor Fusion

Sensor fusion technology is centered on offsetting the limitations of individual sensors to boost the overall system performance by merging data from multiple sensors to deliver a more precise and reliable output than any single sensor alone. This method maximizes the strengths of different sensors while mitigating their weaknesses, resulting in enhanced measurement accuracy, improved stability, and greater situational awareness across various applications.

### 3.1. Levels of Sensor Fusion

Data-level fusion occurs right after the collection of sensor data. This phase involves the integration of raw data from various sensor sources even before any filtering or analytical processes are applied. The primary objective of sensor-level fusion is to process raw data to improve accuracy, diminish noise, and improve the overall data quality. Common techniques employed at this level include the Kalman filter, the complement filter, and the weighted average. Data-level fusion allows for a deeper comprehension of the observed process by combining raw data from different sensors, making it suitable for scenarios where initial filtering and noise reduction are imperative [32].

At the feature-level fusion, the data have already undergone preprocessing and filtering, and key features have been extracted from the raw data through various techniques. These features are amalgamated into a generalized feature vector, offering a more thorough representation of the process. The aim of this level is to minimize data redundancy and dimensionality while retaining essential information. Principal Component Analysis (PCA), Factor Analysis, and Dimensionality Reduction Methods like Multidimensional Scaling (MDS) are the main techniques used for feature-level fusion. Feature fusion enables the optimization and streamlining of the classification process, which is particularly beneficial in applications with limited computational resources [32].

**Figure 2:**
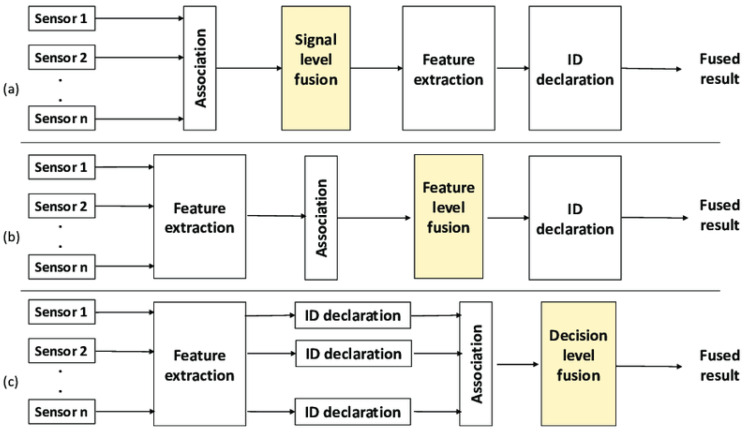
Data fusion at three different levels: (**a**) Signal-level fusion, (**b**) feature-level fusion, and (**c**) decision-level fusion [32].

Decision-level fusion is a level of data fusion in which decisions made by independent classifiers are combined to produce a result. The decision level already contains predictions or classifications from several models, which are combined using methods such as majority voting, Bayesian networks, Dempster–Shafer theory, and ensemble classification methods (e.g., boosting and bagging). The main advantage of decision-level fusion is that it improves the accuracy and robustness of the final classification as it compensates for errors made by individual classifiers. This level is used in situations where robustness to errors and high decision accuracy are important, especially when training data are insufficient, or uncertainty is high. Figure 2 shows the levels of sensory signal fusion and their structural organization [32].

The classification of the sensor fusion levels and their descriptions are provided in Table 1. In a multilevel data-processing system, the information goes through a series of stages, from cleaning the source data and identifying key characteristics (Level 0) to determining the presence and classification of objects, as well as tracking their status (Level 1). The system then analyzes the situation, identifying patterns and synthesizing the data to form a holistic view (Level 2), after which, it predicts possible consequences and assesses risks (Level 3). The final stages optimize the data collection and processing process (Level 4) and establish interactions with the user to improve the system’s understanding and decision making (Level 5) [29].

Table 2 shows the methods for sensor data fusion and its applications in various fields. This overview shows the development of data fusion technology and its contribution to improving system performance.

The methods described in the study range from classical algorithms that are effective in navigation and tracking, such as Kalman filters, to modern methods of machine learning that can handle complex nonlinear data. Deep learning methods can extract high-level features from raw sensor data, which is especially important for autonomous vehicles and environmental monitoring.

Sensor data fusion plays an important role in robotics by improving perception and differentiation, allowing for efficient navigation and interaction. In autonomous vehicles, the combination of radar and camera data improves the accuracy of environmental perception and safety. In environmental monitoring, data fusion improves the accuracy and reliability of the data collected from different sensor networks that are needed to detect changes and make informed decisions. Advanced data fusion algorithms can improve the performance of wireless sensor networks. Special methods can improve navigation in microrobotics [34]. In microelectromechanical systems [35], data fusion algorithms are used for monitoring and tracking. Finally, in the Industrial Internet of Things (IIoT) and equipment fault diagnosis, data fusion methods are used to evaluate efficiency and accuracy. Deep learning methods [36] are widely used in environmental sensor data analysis and autonomous systems.

**Table 2:** Methods and application of sensor fusion.

Methods of Sensor Data Fusion	Application	Achievement	Ref.
Fuzzy logic-based data fusion method	Robotics and automation	Introduced a novel fuzzy logic-based sensor data fusion method	[37]
Sensor data fusion for microrobot navigation	Microrobot navigation	Proposed a method for improving navigation in microrobots through sensor data fusion	[34]
MEMS sensor data fusion algorithms	Micro-electro-mechanical systems	Developed data fusion algorithms for MEMS sensors	[35]
Kalman filters	Navigation and tracking systems	Demonstrated effectiveness of Kalman filters in sensor data fusion	[38]
Deep learning-based sensor data fusion	Environmental monitoring	Introduced a deep learning approach to sensor data fusion for environmental data	[39]
Machine learning-based data fusion	Environmental monitoring	Proposed a multi-sensor data fusion method that uses machine learning	[40]
Deep learning-based multi-sensor data fusion	Autonomous vehicles	Improved sensor data fusion in autonomous vehicles using deep learning	[36]
Survey of sensor data fusion methods	Autonomous driving	Performed comprehensive survey on sensor data fusion methods in autonomous driving	[41]
Sensor data fusion techniques for IoT	Industrial Internet of Things	Discussed sensor data fusion methods that are applicable to IoT environments	[42]

An important trend is that artificial intelligence and deep learning are becoming increasingly integrated into sensor data fusion. This trend is driven by the need to extract large amounts of data efficiently and identify meaningful patterns that traditional methods may miss. In addition, the use of machine learning-based methods shows that the application complexity is increasing, and that more intelligent and autonomous systems are needed. Focusing on applications such as the Internet of Things shows the importance of sensor fusion in establishing a system of interconnection. As industries move towards more connected and intelligent operations, the accurate integration of sensor data is essential for real-time monitoring, predictive maintenance, and overall operational efficiency.

On the other hand, implementing machine learning technique requires multiple trials and therefore takes time if the trials use real machines to learn feedback. In the case of simulating the training of collected datasets, it can lead to uncertainties and cannot be assumed to be accurate, and therefore the reliability still requires some special attention.

### 3.2. Sensor Fusion Software

There are specific software tools for measuring the performance of sensor fusion. This section provides a brief overview of the existing software for sensor data fusion, comparing the different solutions by functionalities, supported operating systems, achievements, and current technology. Table 3 shows the software for the fusion of sensor data, which reflects the diverse applications and requirements of modern engineering and science. The selection of tools depends on the specific requirements of the project, such as the type of sensor used, the performance requirements, and the availability of technical support [35,36,37,38,39,40,41,42,43,44].

Currently, the Robot Operating System (ROS) is a popular tool in the field of robotics. It provides a modular architecture and a wide range of packages for integrating and fusing data from various sensors, which ensures scalability for complex systems [43]. Nevertheless, implementing ROS is difficult for inexperienced users; therefore, the implementation is still limited. The graphical mode of ROS is not very user-friendly.

In addition to ROS, the MATLAB Sensor Fusion and Tracking Toolbox offers tools for modeling, simulating, and implementing data fusion algorithms. Moreover, it can be integrated with other MATLAB tools, making it a versatile solution [44].

Specialized platforms such as Autoware and Apollo are focused on autonomous driving and provide a full set of tools to process and fusing data from LiDAR, cameras, and radar. It is important to note that these platforms are actively developed and are used in industry to create autonomous vehicles.

Similarly, in the field of drones and unmanned aerial vehicles, PX4 Autopilot provides solutions for navigation and flight control. In addition to this, this platform includes optimized data fusion algorithms for real-time operations [49].

Finally, the ETH Zurich Multi-Sensor Fusion Framework tools and libraries provide platforms for experimenting with new data fusion methods, thereby contributing to the development of advanced technologies in the field of navigation.

The overview of the simulation software has revealed that all the software are very versatile and require individual approaches as well as individual operations for each software package. Commercial packages look more polished in terms of the user interface, but open-source ones have much more versatility for development and the implementation of users’ own code.

## 4. Sensor Data Fusion in Technical Applications

### 4.1. Sensor Fusion in Mobile Robotics

**Figure 3:**
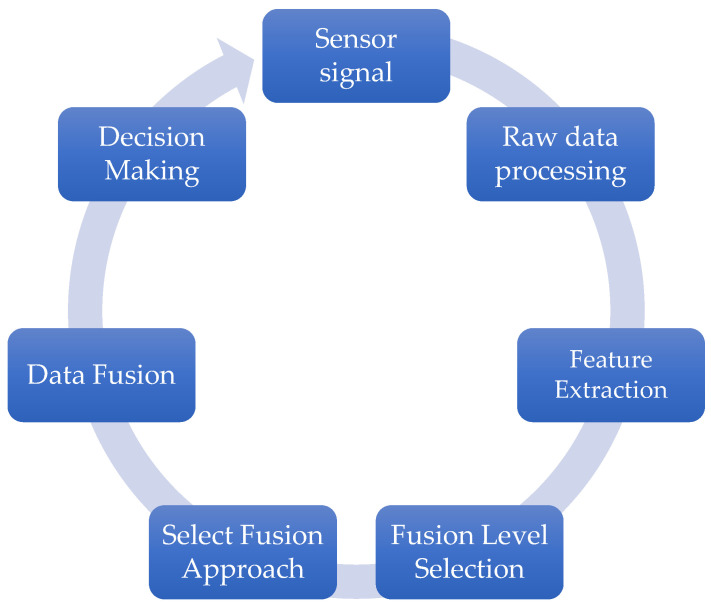
Data fusion in mobile robotics.

In recent years, autonomous mobile robots (AMRs) have become increasingly popular and have been applied in industries, domestic industries, agriculture, medical care, etc. They are capable of autonomous navigation and obstacle prevention and are useful for tasks such as heavy object transport, monitoring, search, and rescue. In the development of AMRs, the main problems include navigation, trajectory planning, and collision avoidance. High accuracy robot localization needs reliable navigation, an understanding of the route to the target point, and the ability to avoid collisions. To increase localization accuracy, relative and absolute methods based on various sensor technologies and algorithms are needed. A prerequisite for AMR functionality is the ability to detect and bypass obstacles on the way to the designated goal. Among the algorithms that are used are bug algorithms, vector field maps (VFHs), and hybrid navigation algorithms, which facilitate the selection of safe routes and the prevention of collisions. To improve accuracy, AMRs use different sensors and data fusion techniques. Sensors are divided into IMUs, monocular vision sensors, and marker-based systems. Data fusion allows for the integration of information from different sensors, which increases the reliability and accuracy of environmental assessments. Ref. [54] presents data fusion methods such as the Kalman filter and particle filter, which are used to process sensor readings. The use of data fusion can improve the accuracy and reliability of AMR systems. Figure 3 illustrates the processing of the classic sensor fusion scheme in mobile robotics.

Autonomous mobile robots (AMRs) are becoming increasingly common in various industries due to their ability to autonomously navigate and avoid obstacles, making them indispensable for tasks that require movement, monitoring, and sensing. However, the development of effective AMRs poses a number of challenges, including precise localization, reliable path planning, and safe obstacle avoidance. To address these challenges, various localization methods, navigation algorithms, and sensor systems are used, as well as data fusion technologies such as Kalman filter and particle filter, to improve the accuracy and reliability of robots in challenging environments.

### 4.2. Sensor Fusion in Production Processes

Sensory data provides “feedback” about the environment and processes. Sensors provide object location and state determination for real-time control, as well as responses to unexpected situations and safety [55]. This allows for greater accuracy and reliability for production processes. Combining data from different sensors allows for a more complete and reliable assessment of the state of the system and the environment, thereby reducing errors and increasing production accuracy. This method is suitable for four specific types of errors; other errors cannot be corrected using this method. Industrial robots require flexible decision making to successfully operate in dynamic and unpredictable environments. Fused sensor data provide more details, allowing control algorithms to respond quickly to changes and effectively adapt robot behavior. The fusion of sensory information helps automate quality control, reduce the risk of defects, and reduce downtime. Robotics standards pay special attention to the quality of sensor systems and data-processing methods. In the development of new standards (for example, the ISO/TC 299 “Robotics” standards [56]), one area is the standardization of the exchange and processing of sensor data for industrial robots.

A framework for combining data from different sensors used to monitor CNC machines is presented in [57]. The aim of this work was to create a more complete and accurate picture of the material processing process by combining information obtained from different sensors. The authors proposed data integration methods that take into account differences in sensor types and the characteristics of the measured parameters. The authors in [58] showed the possibility of using multispectral analysis to measure defects in additive manufacturing. The fusion of data from different spectral ranges allowed users to obtain more complete information about the structure and properties of the material, which is important for defect detection. The authors demonstrated how the fusion of data from different sensors improves accuracy and reliability. Ref. [59] is devoted to the development of a methodology for the multi-sensory monitoring of metal additive manufacturing processes and shows the importance of merging data from different sensors to control the quality of processes. Improving the monitoring quality and management of these processes through multi-sensory methodologies still has limitations. The proposed methodology includes data integration methods that takes into account differences in the types of sensors in robotic manufacturing and the characteristics of the measured parameters. The fusion of optical and feedback sensor data enables real-time monitoring of the robotic welding process and parameter adjustments to achieve stability [60]. The application of optical measurements and feedback can also improve the stability of additive manufacturing based on gas tungsten arc (GTA) robotic welding.

Ref. [61] studied real-time optical monitoring methods in fiber laser welding. The authors analyzed various approaches for the use of optical sensors and emphasized the importance of using data fusion to obtain a more complete picture of the welding process. In [62], the authors applied machine learning to determine the penetration depth across the weld pool in a robotic process. When data from sensors measuring weld pool parameters were merged, the accuracy of the weld penetration depth determination was improved. Using deep learning algorithms for processing sensor output data with raw signals requires a large amount of data and high computational power, and is limited by the high cost of the system. Ref. [63] presents methods for detecting welding defects in high-power disk automatic laser welding based on multi-sensory data analysis. The authors demonstrated how the fusion of data from different sensors revealed hidden patterns and detected welding defects.

The analysis of equipment and process condition monitoring in [64] provided reviews of equipment and process-monitoring methods using sensors, feature extraction techniques from received signals, and artificial intelligence-based monitoring models to classify the condition of the instrument. In particular, the authors considered tool-condition-monitoring methods in milling processes. Ref. [65] used temperature for online monitoring of gear system conditions and provides systematic information on modern monitoring methods and their application in various industries. For full-scale systems, it is practically impossible to obtain adequately similar conditions, so such a model is needed to maintain the operating conditions.

### 4.3. Sensor Fusion Algorithm in Robotics

There is a vast number of position error compensation methods that help to bypass sensor errors and compensate for the positioning error of the microrobotic system.

**Figure 4:**
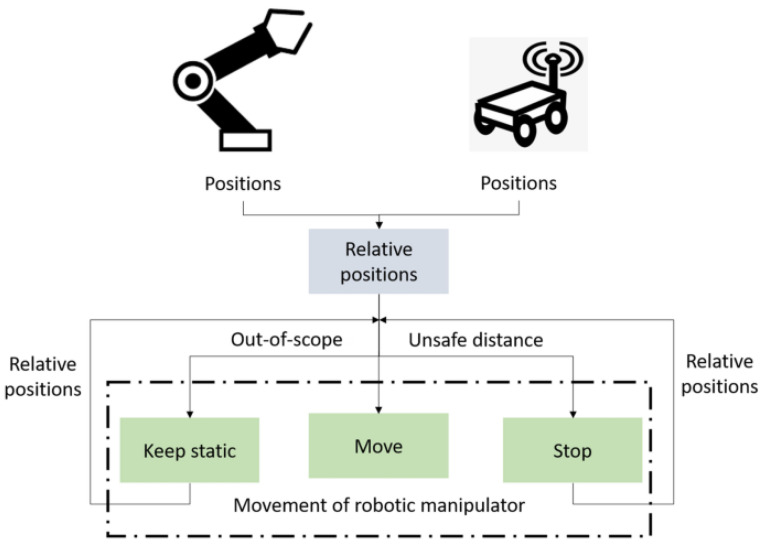
The interaction mode (mobile robot and robotic manipulator) [66].

Ref. [66] presents a continuous part feeding system for industrial manipulators using a mobile robot, a combination of ultrasonic sensors with IMUs, Kalman filter processing, and visual marker detection. The proposed system had high accuracy and reliability for the positioning of the mobile robot, even under dynamic motion and possible blocking of the visual sensor, due to the switching strategy between positioning methods. With the EKF approach, a slight deviation was observed during complex movements, which needed to be compensated for. Figure 4 graphically illustrates the structure and operation of this system.

Various sensor fusion algorithms used for navigation applications have been reviewed, with a focus on MEMS sensors, which are prone to temperature drift and errors. Real-time sensor fusion is essential for low-cost MEMS navigation systems to minimize errors and improve performance [67]. In dynamic experiments, the algorithm only works in rectilinear motions since the relative angle of rotation of the compass is inaccurate. An adaptive fusion algorithm was proposed for real-time processing, integrating data from a gyroscope and accelerometer. This algorithm was designed to improve the estimate of attitude and direction. The authors in [39] reviewed and evaluated the recent research on multimodal imaging sensor calibration for sensor fusion, focusing on both traditional and emerging methods.

The research in [68] aimed to reduce sensor drift errors and improve the accuracy of navigation systems by combining data from redundant MEMS inertial sensors. The results showed that the drift error in a single gyroscope can be significantly reduced by combining the redundant measurements. This method, which decomposes signals into different noise components, requires complex experimental equipment which can introduce new errors.

We designed and implemented an Attitude and Heading Reference System (AHRS) using an extended Kalman filter based on MEMS multi-sensory fusion. This approach improved the accuracy of the measurements and achieved accurate attitude positioning [41]. Ref. [69] focused on the application of multiscale and multi-sensor data fusion algorithms in processing data from MEMS gyroscopes. The fusion algorithm enhanced the reliability, fault detection, and isolation capabilities of the data-processing system. The main problem was the inherent random noise and drift of MEMS gyroscopes, which affected the stability of the entire system. The proposed algorithm based on information fusion was designed to overcome some of these shortcomings by simultaneously filtering the signals.

The alternative sensor fusion method for unmanned aerial vehicle orientation using low-cost MEMS inertial sensors is described in [70]. The authors focused on the correlation between the angles derived from a rate gyroscope and accelerometer-derived angles. The measurements in the study were performed on a flat surface with minimal vibrations since magnetic fields may be degraded by significant vibrations or interference.

A real-time navigation system for a wheeled mobile robot (WMR) using an IMU sensor and Global Positioning System (GPS) modules designed with a real-time trajectory tracking system was developed. This system implements two data fusion methods for localization on an Arduino-based sensor system. The system includes on-chip self-calibration to enhancing its accuracy and reliability in farm conditions. The authors implemented a tracking controller and an embedded system [71]. The main weaknesses identified in the data sources were filtering problems, which are often encountered in real-world settings.

A Gesture Recognition System was developed for robot control using median filtering to reduce noise and sensor data processing with quaternion-based output computing, and employs a dynamic link library for sensor data fusion. The system uses 14 MEMS sensors and Bluetooth for data transmission [72]. Based on a low-cost, intelligent, and lightweight portable gait analysis platform, a body sensor network was developed to assess rehabilitation of patients with gait impairments. The authors used a multi-sensory data fusion algorithm to estimate gait parameters [34]. Table 4 shows the sensor data fusion applications and techniques that have been implemented in different fields.

**Figure 5:**
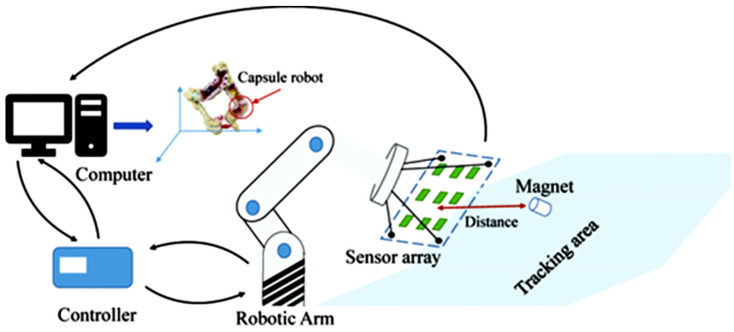
Overview of dynamic magnetic tracking system [75].

Recent research on sensor fusion for robot positioning has developed various innovative approaches to improve positioning accuracy. The classified levels of sensor fusion reflects the complexity and power of signal processing. Many studies integrated multiple sensor types, such as Ultra-Wideband Positioning (UWB) [76], IMUs, cameras, magnetic sensors, and LiDAR. Advanced fusion techniques like the EKF [79], unscented Kalman filter (UKF) [80], and decentralized Kalman filter (DKF) have also been used to improve the control of microrobots and mobile robots in complex environments. For example, some studies [74] utilized diamagnetic levitation and magnetic control to precisely manipulate microrobots without contact, achieving nano-accuracy, which is suitable for biomedical applications. An example of a sensor fusion array is shown in Figure 5.

The applications range from medical environments, where pressure and magnetic sensors improve microrobot stability and navigation in blood vessels, to industrial settings, where sensor fusion improves multirobot formations. Other research used Kalman filters and dedicated algorithms for indoor drone and mobile robot positioning, achieving centimeter-level precision by fusing data from UWB, MEMS sensors, and visual-inertial odometry.

In more specific applications, laser sensors are used to control the closed-loop movement of microrobots and multiple-sensor SLAM technology is used to navigate structured indoor environments [84]. Some approaches integrate sensor data in real time using decentralized algorithms to provide robust and scalable solutions for complex multirobot systems [86]. Decentralized approaches can perform localization even if one or more parts fail, which is a significant advantage for this type of system. Furthermore, fusion techniques such as early integration algorithms combine inertial and visual data to improve GPS-free positioning accuracy, while camera and infrared sensor fusion can be used for high-precision indoor positioning. In general, these studies show the critical role of sensor fusion methods in improving the accuracy and reliability of robotic systems in various fields [87]. The use of IMUs relies on the dead reckoning technique, which is inherently prone to error accumulation, and there are time delays between the camera and IMUs, requiring synchronization.

A multi-sensory fusion-based mobile robot positioning and navigation system is described in [88]. The authors increased the localization accuracy by combining data from inertial measurement units, laser rangefinders, and cameras using an extended Kalman filter. Their findings showed a notable decrease in positioning errors compared to single-sensor systems, improving the robot’s precision in navigating intricate surroundings. Even with a simple trajectory and a short distance, the robot’s performance was still not sufficient to function in more complex conditions.

### 4.4. Sensor Fusion Technique for Localization and Position Detection

In [89], a robust and affordable multiple-sensor data fusion technology for collaborative heterogeneous multirobot positioning is described. The proposed method enables the sharing and fusion of data among robots with different sensor capabilities, thus increasing the overall positioning accuracy. The results demonstrated that this collaborative method improved the positioning efficiency by reducing localization errors and confirmed its usefulness in multirobot systems and remote sensing applications. Achieving high-precision navigation is a challenging task due to the combined effects of various factors, individual sensor limitations, the complexity of the data, and the accumulation of errors.

Other researchers used methods to integrate data from infrared sensors and cameras for indoor positioning using multi-sensory fusion [81]. This approach improved the location accuracy under dynamic conditions by addressing environmental uncertainty and sensor noise. Comparisons between this strategy and those using sensors revealed significant reductions in positioning errors. This study confirmed the practicality and scalability of this approach for large-scale applications due to the low cost of the components and its high accuracy. The camera and infrared sensor integration system still needs to be improved.

In [90], we reported an indoor mapping method based on a combination of IMUs, odometry sensors, and the extended Kalman filter. By intelligently combining data, the localization accuracy in an indoor area was improved without using GPS. The experimental results showed improved accuracy in position estimation; the theoretical calculations showed a good solution to internal navigation problems. When this method was used to estimate the robot position using data from wheel encoders (odometry), a large error occurred both in position and angle. This was due to the accumulation of errors from the encoders.

Similar research [91] used sensor fusion to investigate the location of robotic devices during nuclear reactor inspections. The authors combined data from an IMU and laser rangefinder to increase the accuracy of the robot’s positioning. The test reduced the cumulative positioning errors by less than 2.3 mm and improved the robot’s ability to navigate hazardous areas. The sources of positioning errors included unprocessed laser measurement errors, IMU measurement errors, and scanning resolution interpolation errors. These errors cannot be avoided, and can only be compensated for.

Motion control of a microrobot using laser sensors was implemented in [92]. By fusing the data from laser displacement sensors, the authors achieved precise movement control. The experiments demonstrated that the positioning accuracy was within micrometers for high-precision micro-assembling and manipulation tasks, such as semiconductor and biomedical device manufacturing. The platform was intended for performing movements with a resolution on the order of micrometers. From a practical point of view, the resolution of the movement is limited by the electronic control modules and the uneven distribution of the factor of thirds.

In a similar case [93], scientists unveiled the Micro-UFO (Untethered Floating Object) method, a remarkably precise method of manipulating microrobots. They were able to attain submicrometric accuracy and fine control by employing sensor fusion. Their results demonstrated the efficiency of their manipulation technology and showed potential uses in medical procedures and micromanufacturing, where great precision is critical. Thus, despite the high positioning accuracy, the system has limitations related to hydrodynamic forces, phase differences, trajectory errors during complex movements, and a stable levitation range.

Research was conducted on the use of pressure signals in pulsating blood vascular flow to stabilize the location of microrobots. They were able to maintain the stability of the microrobot in dynamic biological contexts by combining data from sensors. Their findings suggested that the microrobot can be successfully stabilized within a pulsatile flow and that it could be used in targeted therapies and minimally invasive medical procedures [94]. The hemorheological properties of blood and blood flow in the vascular system create significant hydrodynamic obstacles for the control of microrobots. The high velocity of blood flow, especially in arteries, makes it difficult to precisely guide microrobots, whose speed is actually much lower.

In [95], the authors present a two-probe setup and developed algorithms for the precise capture, movement, and placement of nanoparticles using visual feedback from a microscope and sensory data from the probes. The experimental results showed that system can work with position errors of less than 5 micrometers, which is essential for tasks requiring high precision and responsiveness in the fields of microfabrication. Although the system, in principle, has a high resolution, the accuracy of the image data deteriorated significantly when the two probes were close to each other.

The results demonstrated that the multi-sensory fusion-based mobile robot positioning algorithm increased the positioning accuracy. Their algorithm effectively reduced location errors by combining data from IMUs, LiDAR, and visual sensors. The results showed that the accuracy and reliability were improved, addressing the difficulties of localization which are essential for autonomous navigation [96]. In a complex scenario with a glazed corridor and an exterior corridor, cartographer showed a noticeable angular deviation and a dependence on the quality of the LiDAR data.

A pre-integration technique for inertial navigation that uses multi-sensory fusion for the location of indoor mobility robots to improve the accuracy of inertial navigation was proposed. The results showed that the drift was significantly reduced over time, making it useful for indoor navigation when GPS signals are weak or opacity is lacking. This approach improved the robot’s ability to maintain accurate positioning over a long period of time [97]. Although various sensors have been used to detect key points, some of them may have limitations. For example, a Harris corner may not work with sharp changes in scale or significant rotation between image frames.

Range-visual-inertial sensor fusion was used for the localization and navigation of micro-aerial vehicles. Combining range measurements with visual and inertial data improved the localization accuracy of micro-aerial vehicles. The experiments demonstrated improved robustness and accuracy in complex air operations, while maintaining reliable positioning in environments with limited functions or GPS rejection [98]. The experiments used minimally tuned standard PID controllers, which could affect the accuracy of trajectory tracking, especially at higher speeds.

Ref. [76] studied the formation of multiple robots with sensor locations in unknown environments. Using data from LiDAR, IMU, and inter-robot communication, the robots were able to maintain training and conduct collaborative navigation without prior environmental knowledge. Their results showed that training control was successful with minimal position errors and demonstrated the effectiveness of their approach in unrecognized and dynamic terrains. During trajectory tracking in real-world conditions, the robots exhibited slight vibrations while moving along a defined path due to localization errors.

In [99], a sensor fusion system was developed to autonomously locate mobile robots. The proposed system improved the autonomy and precision of robot positioning. The results showed higher reliability and reduced the positioning errors compared to GPS alone, contributing to the development of a fully autonomous robot system capable of operating in various environments. This paper discusses methods for combining information from laser scanners and cameras to improve terrain perception by robots. The authors proposed a new approach using deep neural networks and conditional random fields. The excessive complexity of the deep structure of the model will lead to additional loads in the learning network.

A new micro-profilometric method was developed for the study of art objects by combining intensity and surface height data obtained from interferometric sensors. Ref. [100] describes the principle of this system, and the testing of its characteristics and practical application in the field of cultural heritage conservation. The disadvantage of the method is that it is often impossible to obtain high-quality data in a single scan due to the different light absorption coefficients of different materials.

Ref. [101] presents a method for positioning mobile robots based on multi-sensory information fusion with laser SLAM. Integrating laser scanning data with IMU and odometer data, the authors improved the positioning accuracy and map quality. Their results showed that the localization errors were reduced and the environment was mapped more accurately, which is important for autonomous navigation and environmental interactions, although the high complexity of the algorithm and computational workloads limits implementing of this method.

The positioning of a wheeled mobile robot based on multi-sensory data fusion using the extended Kalman filter is described in [102]. By combining data from an IMU, wheel encoder, and ultrasonic sensor, the reliability of the mobile robot positioning system was increased. Their experiments showed that the cumulative error rate was significantly reduced over time and that the robot’s ability to navigate accurately over longer distances and in environments with sensor uncertainty improved. There are numerous signal-filtering techniques that are well studied and well known. However, each signal has its own footprint with specific features, so filtering issues are still important in technology and are part of the sensor fusion process. Mobile robots can define their orientation and coordinates using a minimum number of sensors when the sensor fusion mechanism is applied [103]. Unfortunately, the planning of the optimal robot route in dynamic environments, especially with unknown and unpredictable changes, becomes a difficult task.

In general, the presented studies show the importance and effectiveness of multi-sensory data fusion methods in improving the accuracy and reliability of positioning in various robotic systems, from microrobots to mobile platforms and unmanned aerial vehicles. These results open broad prospects for the further development of autonomous systems and their application in various fields, including medicine, manufacturing, and navigation in complex environments.

## 5. Discussion

After an analysis of the existing sensor data fusion issues, the authors noticed some trends. The distribution of sensor fusion technology in each area of application is presented in Table 5.

Autonomous vehicles and drones actively use all levels of data fusion, especially hybrid fusion, to ensure reliable operation under dynamic conditions.

Service robots and medical robots also require a high level of data integration, especially at the high-level and hybrid levels, to ensure effective interactions with people and the performance of complex tasks.

**Figure 6:**
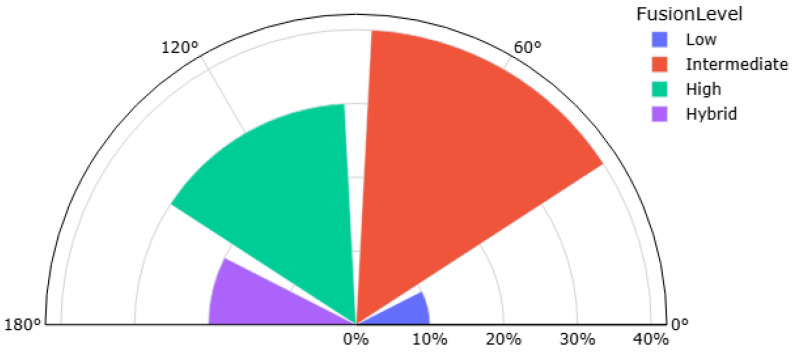
Graphical classification of sensor fusion technologies in the field of robotics.

Industrial robots are less dependent on high-level data fusion as they usually perform repetitive tasks under predictable conditions, which reduces the need for complex processing of sensor data. A graphical classification of sensor fusion is shown in Figure 6.

The analysis of data fusion technology revealed that the intermediate level accounts for a higher percentage of all sensor data fusion implementation, and could therefore open a new space for higher level technique development. It is also necessary to develop more sophisticated algorithms that can effectively process and interpret the combined data to make more accurate and timely decisions. This will lead to the improved performance and reliability of systems based on sensor data fusion.

## 6. Conclusions

This review identified numerous studies devoted to the use of sensor data fusion to improve the accuracy and reliability of the positioning and navigation of robotic systems. The use of multi-sensory fusion can effectively compensate for the disadvantages of individual sensors, improving the quality of the system. Some publications focused on the combination of raw data from different sensors. For example, integrating data from IMUs, odometry, and laser rangefinders can improve positioning accuracy by directly combining the measurements. Such a fusion requires precise data synchronization and differences in the update rate and characteristics of the different sensors. Several studies focused on combining extracted features from sensor data, for example, combining visual features with LiDAR data to improve mapping and localization in SLAM systems. This approach uses complex information from different types of sensors to more reliably recognize the environment. Fusion is also used at the decision-making level, where the results of individual algorithms or models are combined to make a final decision, for example, in systems where data from different robots are combined for joint navigation and formation.

Several studies used a hybrid fusion level, combining low-, middle-, or high-level fusion methods to achieve the best results. This approach has the advantage of using each fusion level simultaneously. In hybrid methods, the data from different sensors are first combined at the data or feature level, and then the results are integrated at the decision level. This helps improve the accuracy and reliability of systems in complex and dynamic environments.

Future research in this area could focus on developing adaptive fusion methods that can dynamically select the optimal fusion level and method depending on current conditions and tasks. Machine learning and deep learning methods are also promising methods for improving data fusion and decision making in real time, which will allow for the creation of more intelligent and autonomous methods.

The provided analysis of the issues in robotics revealed the following trends:

- The widespread adoption of multi-sensory fusion as a key approach to overcome the limitations of individual sensors;
- A movement towards more complex and adaptive fusion algorithms that can effectively operate in real time and dynamically respond to environmental changes;
- The integration of machine learning and deep learning methods to improve the quality of data fusion and decision making;
- The creation of highly autonomous robotic systems capable of performing complex tasks under uncertain and changing conditions.

These trends reflect the growing interest in the development of intelligent navigation and positioning systems that can find application in various fields, including industry, medicine, and service robotics.

Future trends in hybrid sensor data fusion could include the development of hybrid models of fusion, including sensor-database mixed data fusion and machine learning-based sensor signal fusion with dynamic weight coefficient adjustment. There are AI technologies that can be implemented into fusion processes and there are unpredictable technologies development trends within the hybrid sensor fusion domain.

## Figures and Tables

**Table 1:** Levels of sensor fusion (classification according to the Joint Directors of Laboratories model).

Levels of Fusion	Description	Ref.
Level 0: Sub-Object Data Assessment	Removal of noise and unwanted signals. Correction of systematic measurement errors. Finding key characteristics from data.	[33]
Level 1: Object Assessment	Determination of the presence of objects in data. Determination of the type or category of an object. Monitoring of the position and state of an object over time.	[29]
Level 2: Situation Assessment	Identification of patterns and anomalies in object actions. Assessment of the environment and conditions. Combining the data to create an overall picture of the situation.	[33]
Level 3: Impact Assessment	The system predicts possible consequences of the current situation and assesses risks	[33]
Level 4: Process Refinement	Adaptation and optimization of the data collection and processing based on the results obtained.	[29]
Level 5: User Refinement	Interaction between the data fusion system and the user to improve the system’s understanding and decision making.	[33]

**Table 3:** Sensor fusion software.

Software	OS	Features	Ref.
ROS (Robot Operating System) and ROS 2 (all distrbutions)	Linux macOS Windows	Open platform for robotics Many packages for data fusion (robot_localization, sensor_msgs, etc.) Wide support for sensors and algorithms Large community and active development	[43]
MATLAB Sensor Fusion and Tracking Toolbox R2024b	Windows macOS Linux	Tools and algorithms for multi-sensor fusion Support for object tracking and localization Simulation and scenario testing Used in academic and industrial research	[44]
OpenCV 4.11.0	Linux macOS Windows Android iOS	Computer vision library Offers functions for processing and merging data from cameras and sensors Widely used in image and video processing Large community and extensive documentation	[45]
RTMaps (Real-Time Multi-Sensor applications)	Linux Windows	Platform for real-time and multi-sensor applications Synchronous data acquisition and processing Used in the automotive and robotics industries Graphical development environment	[46]
Autoware (all versions)	Linux	Open-source software for autonomous driving Performs fusion of data from LiDAR, cameras, and radar Based on ROS Used in autonomous vehicle projects	[47]
Apollo (Baidu Autonomous Driving Platform) (all versions)	Linux	Open platform for autonomous driving Performs fusion of data from various sensors Modular architecture Support from major company Baidu	[48]
PX4 Autopilot v1.16.0	NuttX (RTOS) Linux	Open platform for drones and UAV autopilot Offers data fusion algorithms for navigation Large developer community Used in research and commercial UAVs	[49]
LidarView v4.4.0 (by Kitware, Clifton Park, NY, USA)	Windows macOS Linux	Visualization and processing of LiDAR data Provides support for fusion of LiDAR data with data from other sensors Based on VTK and ParaView technologies Used in research and industry	[50]
Bosch Sensor Fusion SDK v3.4.0	Android iOS	Designed for mobile applications Provides data fusion algorithms for motion tracking Used in smartphones and wearables Commercial SDK (by Bosch Sensortec, Reutlingen, Germany)	[51]
Kalman Filter Libraries (TinyEKF, etc.) (all versions)	Any OS (C/C++, Python)	Offers Kalman filter implementations for data fusion Used in embedded systems Provides support for extended and non-linear Kalman filters Lightweight and suitable for systems with limited resources	[52]
FusionLib (all versions)	Windows Linux	Data fusion library for C++ Supports various fusion algorithms Modular architecture for easy integration Has documentation and examples for quick start	[53]
Multi-Sensor Fusion Framework (by ETH Zurich’s) (all versions)	Linux	Unified platform for data fusion Provides support for various types of sensors Modular and extensible Developed at a leading research university	[54]

**Table 4:** Comparison of sensor data fusion applications.

Methodology	Application	Sensors	Fusion Technique	Ref.
Signal level fusion for vibration reduction	Micro assembly	Diverse sensors	Signal-level fusion	[73]
Passive diamagnetic levitation	Microrobot manipulation in fluid environments	Magnetic fields	Magnetic control	[74]
Movable sensor array with dynamic tracking	Medical microrobotics	Magnetic sensors	Multi-point locating algorithm	[75]
Algorithm for multirobot formation	Multirobot formation	Ultrawideband system, IMUs, wheel encoders	Sensor fusion system	[76]
Magnetic control for patterning	Fiber functionalization	Magnetic sensors	Magnetic control	[77]
Laser sensors with feedback and control	Microrobotic motion control	Laser sensors	Closed-loop motion control	[78]
PL-ICP and extended Kalman filter (EKF)	Indoor Robot SLAM	LiDAR, cameras, IMUs, odometers	EKF, Bayesian	[79]
UKF-based sensor fusion	Mobile robot localization	IMUs, angle sensors	UKF algorithm	[80]
Maximum Likelihood Estimator	Indoor positioning	Cameras, infrared sensors	Fusion estimation	[81]
Resilient Factor Graph	Robot navigation	UWB, IMUs, LiDAR	Factor Graph Optimization	[82]
EKF with sensor fusion	Indoor localization of mobile robots	IMUs, odometers, laser radar	EKF	[83]
RBPF-SLAM with Maximum Posterior Estimation	Mobile robot navigation	Laser radar, ultrasonic sensors, monocular cameras	RBPF	[84]
Sensor Fusion with SLAM and LiDAR Scan	Mobile robots	LiDAR, GNSS, IMUs, wheel encoders	Sensor Redundancy Strategy	[85]

**Table 5:** Comparison of sensor data fusion applications according to fusion levels.

Fusion Level	Description	Examples	Applications
Low-level fusion	Directly combines raw data from sensors, focusing on signal-level information.	IMU + GPS for raw positioning; LiDAR + camera for depth estimation.	Basic localization, rough mapping, and obstacle detection.
Intermediate-level fusion	Processes and combines features extracted from raw data for more significant ideas.	Feature-based fusion like parameter detection and object segmentation.	Object tracking, pattern recognition, and enhanced localization.
High-level fusion	Combines high-level decisions made by each sensor, focusing on interpreted or classified data.	Decision fusion for obstacle avoidance or object recognition.	Advanced navigation, autonomous decision making, and robotic manipulation.

## Data Availability

Data are contained within the article.

## Abbreviations

The following abbreviations are used in this manuscript:

AHRS	Attitude and Heading Reference System
AMRs	Autonomous mobile robots
ANFIS	Adaptive Neuro Fuzzy Interface
CNC	Computer numerical control
DKF	Decentralized Kalman filter
EKF	Extended Kalman filter
GTA	Gas tungsten arc
GNSS	Global Navigation Satellite System
IoT	Industrial Internet of Things
IMUs	Inertial measurement units
IR	Infra-red
KF	Kalman filter
LiDAR	Light detection and ranging
LSTM	Long short-term memory
MIMUs	Magnetic and inertial measurement units
µSCM	Micro search-coil magnetometer
MEMS	Microelectromechanical system
HES	Micro-Hall-effect sensor
MDS	Multidimensional Scaling
PDR	Pedestrian dead reckoning
PCA	Principal Component Analysis
RF	Radio frequency
ROS	Robot Operating System
SFAs	Sensor fusion algorithms
SLAM	Simultaneous localization and mapping
UWB	Ultra-Wideband Positioning
UKF	Unscented Kalman filter
UFO	Untethered Floating Object
VFHs	Vector field maps
WMR	Wheeled mobile robot
